# Biomechanical comparison of a novel transoral atlantoaxial anchored cage with established fixation technique - a finite element analysis

**DOI:** 10.1186/s12891-015-0662-7

**Published:** 2015-09-22

**Authors:** Bao-cheng Zhang, Hai-bo Liu, Xian-hua Cai, Zhi-hua Wang, Feng Xu, Hui Kang, Ran Ding, Xiao-qing Luo

**Affiliations:** Department of Orthopaedics, Wuhan General Hospital of Guangzhou Command of PLA, Wuhan 430070, China; Southern Medical University, Guangzhou 510515, China; Institute of Applied Mechanics and Biomedical Engineering, Taiyuan University of Technology, Taiyuan 030024, China; The School of Internet of Things, Jiangnan University, Wuxi 214122, China

## Abstract

**Background:**

The transoral atlantoaxial reduction plate (TARP) fixation has been introduced to achieve reduction, decompression, fixation and fusion of C1–C2 through a transoral-only approach. However, it may also be associated with potential disadvantages, including dysphagia and load shielding of the bone graft. To prevent potential disadvantages related to TARP fixation, a novel transoral atlantoaxial fusion cage with integrated plate (Cage + Plate) device for stabilization of the C1-C2 segment is designed. The aims of the present study were to compare the biomechanical differences between Cage + Plate device and Cage + TARP device for the treatment of basilar invagination (BI) with irreducible atlantoaxial dislocation (IAAD).

**Methods:**

A detailed, nonlinear finite element model (FEM) of the intact upper cervical spine had been developed and validated. Then a FEM of an unstable BI model treated with Cage + Plate fixation, was compared to that with Cage + TARP fixation. All models were subjected to vertical load with pure moments in flexion, extension, lateral bending and axial rotation. Range of motion (ROM) of C1-C2 segment and maximum von Mises Stress of the C2 endplate and bone graft were quantified for the two devices.

**Results:**

Both devices significantly reduced ROM compared with the intact state. In comparison with the Cage + Plate model, the Cage + TARP model reduced the ROM by 82.5 %, 46.2 %, 10.0 % and 74.3 % in flexion, extension, lateral bending, and axial rotation. The Cage + Plate model showed a higher increase stresses on C2 endplate and bone graft than the Cage + TARP model in all motions.

**Conclusions:**

Our results indicate that the novel Cage + Plate device may provide lower biomechanical stability than the Cage + TARP device in flexion, extension, and axial rotation, however, it may reduce stress shielding of the bone graft for successful fusion and minimize the risk of postoperative dysphagia. Clinical trials are now required to validate the reproducibility and advantages of our findings using this anchored cage for the treatment of BI with IAAD.

## Background

Basilar invagination (BI) is characterized by irreducible atlantoaxial dislocation (IAAD) and upward migration of the tip of the odontoid process of C2 vertebra [1]. Basilar invagination is usually secondary to congenital regional malformation [2], or bone diseases such as rheumatoid arthritis, hyperparathyroidism, Paget disease, osteogenesis imperfect and rickets [3]. It results in progressive compression on cervical spinal cord, leading to profound neurologic deficits and even death [4].

The selection of the surgical approach depends mainly on the reducibility of the atlantoaxial dislocation [4–6], as for the cases of BI with IAAD, which can not be reduced in traction before surgery, several authors of clinical studies have shown that transoral anterior atlantoaxial release followed by transoral atlantoaxial reduction plate (TARP) fixation can achieve reduction, decompression, fixation and fusion of C1–C2 through a transoral-only approach [4, 7–9]. Although spacer or cage has been used to reduce the BI through posterior approach [1, 2, 10], there are no studies referred to the cage through transoral approach for C1-C2 fusion. The atlantoaxial fusion cage with TARP (Cage + TARP) device may increase stability and fusion rates, maintain or improve atlantoaxial fusion angle and prevent bone graft collapse, extrusion, resorption and micromotion; however, the Cage + TARP device may also be associated with potential disadvantages and complications, including dysphagia, load shielding of the bone graft.

To prevent potential disadvantages related to Cage + TARP fixation, a transoral atlantoaxial fusion cage with integrated plate (Cage + Plate) device for stabilization of the C1-C2 segment is designed based on the 2-screw anchored cage (COALITION, Globus Medical) [11], which is designed for use following anterior cervical discectomy for reduction and stabilization of lower cervical spine. The Cage + Plate device has a lower profile to minimize the risk of dysphagia and may potentially prevent load shielding of the bone graft. Because the design of the Cage + Plate device and the biomechanics of the C1-2 joint are different from the subaxial cervical implant and the subaxial cervical spine, the biomechanical properties of this anchored cage remain unclear.

The FE analysis is well suited to parameter studies and allows to investigate the stress distribution of the instrumentation than vitro experiment [12]. In 2000, Puttlitz et al. [13] first reported validated FEM of the upper cervical spine (C0-C2), which was used to study the pathology of rheumatoid arthritis in the craniovertebral junction. Since then, the FEM of the C0-C2 complex was developed to simulate the complex kinematics of the upper cervical spine [14, 15] and has already been used widely for analyzing biomechanical of various instrumentation system designed for use in the upper cervical spine [12, 16, 17]. Hence, we built a FEM of the intact upper cervical spine to reproduce physiologic conditions. Then a FEM of the unstable BI model, treated with Cage + Plate fixation,was compared with the Cage + TARP fixation. Using FE analysis, the purpose of our study is to determine the biomechanical difference between the Cage + Plate device and Cage + TARP device at C1-C2 segment and to investigate stress distribution of the two devices under the same condition.

## Methods

Four FEMs of the C0-C2 were reconstructed in our study. FEMs included the intact model, unstable BI model, unstable BI model implanted with either Cage + Plate device or Cage + TARP device at C1-C2 segment.

### Intact model and unstable BI model

**Fig. 1:**
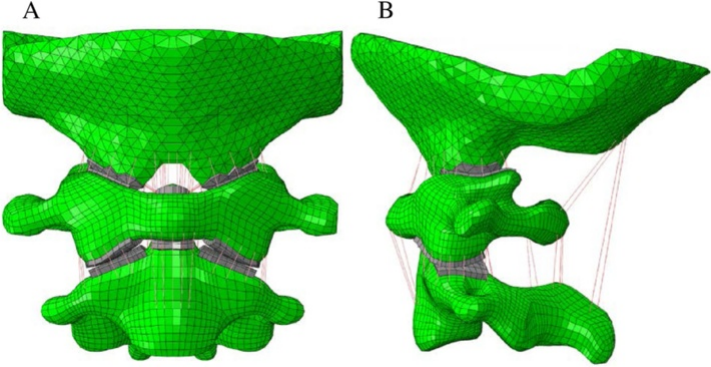
The frontal (**a**) and lateral view (**b**) of FEM of the intact upper cervical spine

To build an FEM, we obtained computed tomography images at 0.5-mm intervals on C0–C2 of a 31-year-old healthy man with a height of 175 cm. Ethics committee approval for use of individual participant data was granted by the ethics committee of Wuhan General Hospital prior to this study. Informed consent in the study was also obtained from the participant. The images were performed for boundary detection by custom-made software. The commercially available finite-element program Abaqus 6.9 (Dassault Systemes, USA) was used to model the C0-C2 complex and to evaluate differences between the Cage + Plate and Cage + TARP for C1-C2 fixation. The FEM of the C0-C2 complex included cortical bone, cancellous bone and cartilage, and 10 types of ligaments (anterior longitudinal ligament, anterior atlanto-occipital membrane, tectorial membrane, posterior atlanto-occipital membrane, posterior atlanto-axial membrane, joint capsules, alar ligament, apical ligament, transverse ligament and cruciate ligament-vertical portion). The transverse ligament is low elastic tissue and quite tough, so it was modeled with 4-node membrane elements [14]. All other ligaments were modeled with linear contact elements applying only to the tension force. The C1-C2 vertebral were defined as an internal cancellous bone core surrounded by a 1.5-mm-thick cortical outer shell, whereas occiput were defined as entire cortical bone. Cortical bone, cancellous bone and cartilage of C1-C2 vertebra were modeled with 8-node isoparametric hexahedral elements, whereas cortical bone of C0 were modeled with 4-node isoparametric tetrahedral elements in this study. Then a three-dimensional non-linear FEM of intact upper cervical spine was created (Fig. 1a-b). The intact model consisted of 26,523 elements and 19,637 nodes. For the unstable BI model, we removed all elements representing transverse ligament from the intant model. In addition, BI with IAAD always had assimilation of C1, with rates up to 92.0 % [18], thus we defined the C0–C1 joint as tie contact to simulate such anatomical abnormality of BI.

Sliding contact definitions with friction were used for the facet joints as well as between the occipital condyles and the atlas, the atlas and the dens, the dens and the transverse ligament, and the atlas and the axis, and the friction coefficient was set at 0.1 [14]. The initial gap between the articulating surfaces was based on computed tomography images. The material properties of the FEM were selected from previous published studies (Table 1) [12, 14, 15, 17].

**Table 1:** Material properties and model characteristics of the Current FEM. C3D4 is the 4-node isoparametric tetrahedral elements, C3D8 is the 8-node isoparametric hexahedral elements, and T3D2 is the Truss Element with Two Nodes in the ABAQUS Software

Components	Element type	Youngs Modulus(MPa)	Poisson’s Ratio
Cortical bone	C3D4	12,000	0.29
	C3D8		
Cancellous bone	C3D8	450	0.29
Cartilages	C3D8	10	0.3
Transverse ligament	4-node membrane elements	20	0.3
Cruciate ligament-vertical portion	T3D2	10	0.3
Alar ligament	T3D2	5	0.3
Apical ligament	T3D2	10	0.3
Anterior longitudinal ligament	T3D2	10	0.3
Anterior atlanto-occipital membrane	T3D2	10	0.3
Tectorial membrane	T3D2	10	0.3
Posterior atlanto-occipital membrane	T3D2	10	0.3
Posterior atlanto-axial membrane	T3D2	10	0.3
Joint capsules	T3D2	10	0.3
Cage	C3D8	3,600	0.25
Bone graft	C3D8	450	0.29
Screw or TARP (Ti-6Al-4 V)	C3D8	110,000	0.3

### FEM of Cage + plate device

The Cage + Plate device is a zero profile, asymmetrical hexagon shaped cage integrated with an anterior titanium plate containing two screws in the same horizontal plane for stabilization of the C1–2 joint: the medial C2 intraarticular screw is designed as one oblique screw from the medial corner of the C2 lateral mass to the C2 pars interarticularis, while the lateral C1 intraarticular screw is an oblique screw from the lateral part of the C1 lateral mass in an anteroposterior direction.

**Fig. 2:**
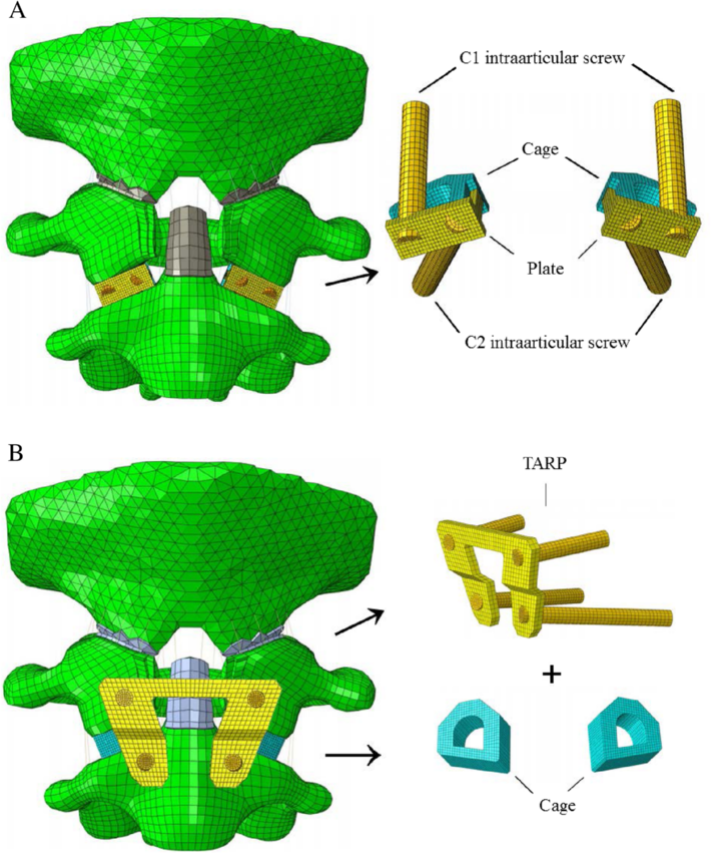
FEMs of the unstable upper cervical spine implanted with two different devices. **a** Cage + Plate fixation device; **b** Cage + TARP fixation device

Important neurovascular structures to be avoided during C1 intraarticular screw and C2 intraarticular screw placement are the spinal cord medially, the vertebral artery (VA) posterolaterally at C1 and C2, and the atlanto-occipital joint cranially. A too-medial trajectory of C2 intraarticular screw may injure the cord, whereas drilling too laterally may injure the VA. For the C1 intraarticular screw, if it is inserted too laterally, it may violate the lateral cortex at the level of C1 transverse foramen or above/below it, leading to VA injury [19]. Therefore, we provided the C1 screw in an anteroposterior direction. It should also prevent C0–C1 joint violation because the C1 lateral masses on both sides have a bow-tie shape in the coronal plane, which result in their height is longer in the lateral part and shorter in the medial part [19]. Overall, the C1 screw can be inserted without C0–C1 joint and VA violation, when its tip trajectory is in an anteroposterior direction and lies below the axial plane of the superior margin of the VA groove of C1. In this study, the trajectory of the intraarticular screw was determined to be 39.2° upward for C1 and 30.0° downward and 12.1° outward for C2, and the length of the intraarticular screws were 19.7 mm for C1 and 36.6 mm for C2, respectively. Finally, the FEM of Cage + Plate was completed (Fig. 2a). The Cage + Plate model consisted of 41,442 elements and 39,943 nodes.

### FEM of Cage + TARP device

The FEM of TARP device was performed as described by Ai and Yin [7]. The operative procedure of TARP has been described in detail elsewhere [4, 7–9]. The entry point of the unicortical C1 anterior lateral mass screw was located 5 mm away from the inner-lower edge of the C1 lateral mass and the entry angle of C1 anterior lateral mass screw was 5°–10° upward and 10°–15° outward along the longitudinal axis of the C1 lateral mass [4]. Unicortical screw was used because bicortical purchase may injure the VA within the VA groove along the C1 posterior arch, as well as the C2 ganglion and venous plexusis, which occupies up to three quarters of the intervertebral space between C1 and C2 [20]. The C2 anterior pedicle screw entry point was 5 mm below the vertex point of medial corner of superior lateral mass of C2, and the entry angle of C2 anterior pedicle screw was 6.5° to 21.5° inferiorly in the saggital plane and 9.3° to 28.3° laterally in the axial plane [21]. In this study, the direction of the anterior screw was determined to be 9.7° upward and 10.1° outward for C1 and 20.1° downward and 14.9° outward for C2, and the length of the anterior screws were 20.3 mm for C1 and mm for 33.2 mm for C2, respectively. Unlike adopting granulated iliac crest autograft [9], we inserted self-designed cage with cancellous bone graft into C1–2 joint. Finally, the FEM of Cage + TARP was completed (Fig. 2b). The Cage + TARP model consisted of 36,788 elements and 34,772 nodes.

### Transoral surgical technique simulations

With the neck in hyperextension and mouth opened wide, the transoral approach generally accesses C0 to C3, and specialized retractors are used to maintain the access for transoral procedure [22]. To achieve ideal reduction and fusion of C1–C2, the cortical bone of the anterior arch of C1, contractural scar tissue between atlas and odontoid, contractural articular capsule, as well as the articular cartilage were removed. Sometimes a high-speed burr, or an angled curette is used to resect the cortex [4, 7–9]. Then a fusion cage filled with bone graft was inserted into the C1-2 joint in a direction parallel to the sagittal plane. After cage placement, a suitable TARP plate was rigidly fixed to screws from C1 to C2 to provide additional stability for fusion. For the Cage + Plate device, two diverging intraarticular screws were constructed from titanium plate, which was integrated with the self-designed cage. The specialized screwdrivers may be used to implant screws with bending angle for Cage + Plate device.

### Interactions, loading and boundary conditions

In this FE study, the micromotion between the bone and screw was ignored, and the interface between screw and surrounding bone was defined as tie contact condition without movement. A higher friction coefficient of 0.8 was applied to the interface of the bone and cage after the surgical procedure in the implanted models [23].

All nodal points of the lower end plate of C2 vertebra were fixed in all degrees of freedom, while the C0 of FEM was subjected to a pure moment of 1.5 Nm in flexion, extension, lateral bending, and axial rotation. The FEM was also subjected to vertical load of 40 N applied on the C0 to simulate head weight as suggested by Puttlitz et al. [16]. Range of motion (ROM) of C1-C2 segment and maximum von Mises Stress (MVMS) of the C2 endplate and bone graft were quantified for the two devices

## Results

### Validation of intact model and unstable BI model

To validate our model, the ROM of the C0-C1 and C1-C2 segments was calculated and compared with the results of in vitro tests performed by Panjabi and Dvorak et al. [24] and Panjabi et al., [25, 26] as well as the results of in FE tests performed by Zhang et al. [15]. We found good agreement between our results and previously published data (Table 2). The unstable BI model increased ROM of C1-C2 segment in flexion, extension, lateral bending, and axial rotation by 35.2 %, 16.4 %, 4.0 %, and 5.6 %, respectively, as compared with the intact model (Table 2). Based on these findings, we confirmed the validity of our intact model as well as the unstable BI model.

**Table 2:** Results From Validation of the FEM of the Upper Cervical Spine (°)

Load case	Joint	Panjabi et al. [24]	Panjabi et al. [25, 26]	Zhang et al. [15]	Intact model	Unstable BI model
Flexion	C0-C1	3.5 ± 0.6	10.8–17.2	14.5	12.5	-
	C1-C2	11.5 ± 2.0	9.8–16.2	15.0	12.5	16.9
Extension	C0-C1	21.9 ± 1.9	10.8–17.2	13.3	16.7	-
	C1-C2	10.9 ± 1.1	6.0–16.0	12.7	12.2	14.2
Lateral bending	C0-C1	5.6 ± 0.7	2.6–8.6	5.5	3.7	-
	C1-C2	4.0 ± 0.8	3.8–19.6	5.9	5.0	5.2
Axial rotation	C0-C1	7.9 ± 0.6	1.0–10.5	8.5	8.3	-
	C1-C2	38.3 ± 1.7	24.2–46.4	30.6	28.5	30.1

### ROM at the C1-C2 level

Both Cage + Plate and Cage + TARP models significantly reduced ROM compared with the intact state. The Cage + Plate model reduced the ROM at the C1-C2 level by 96.8 %, 94.7 %, 98.0 % and 98.8 % in flexion, extension, lateral bending, and axial rotation, respectively, compared with the intact model. The Cage + TARP model reduced the ROM at the C1-C2 level by 99.4 %, 97.1 %, 98.2 % and 99.7 % in flexion, extension, lateral bending, and axial rotation, respectively, compared with the intact model. The Cage + TARP model reduced the ROM by 82.5 %, 46.2 %, 10.0 % and 74.3 % in flexion, extension, lateral bending, and axial rotation, respectively, compared with Cage + Plate model (Fig. 4). This indicates that the Cage + Plate device may provide similar stability in lateral bending but lower stability in flexion, extension, and axial rotation in comparison to the Cage + TARP device.

### Stress of the C2 endplate

Since cage subsidence most commonly occurs at the upper endplate of the lower vertebra at the operated segment [27], only the maximum stress on the C2 endplate was calculated. The C2 endplate of the Cage + Plate model had higher stress than that of Cage + TARP model in all motions (Fig. 5a-d). The ratios of MVMS at the C2 endplate in Cage + TARP and Cage + Plate models were 1: 8.3 in flexion, 1: 2.2 in extension, 1: 2.5 in lateral bending, and 1: 2.9 in axial rotation, respectively. The MVMS at the C2 endplate in both models was found in extension: 32.4 MPa occurred in Cage + Plate model and 14.9 MPa in Cage + TARP model.

### Stress of the bone graft

The MVMS of the bone graft was found to be high in Cage + Plate device in all motions (Fig. 6). The ratios of MVMS at the bone graft in Cage + TARP and Cage + Plate models were 1: 107.5 in flexion, 1: 25.3 in extension, 1: 36.3 in lateral bending, and 1: 31.3 in axial rotation, respectively. This indicates that in comparison with Cage + TARP device, the Cage + Plate device significantly reduces stress shielding of the bone graft.

## Discussion

### Design of the transoral atlantoaxial fusion cage

**Fig. 3:**
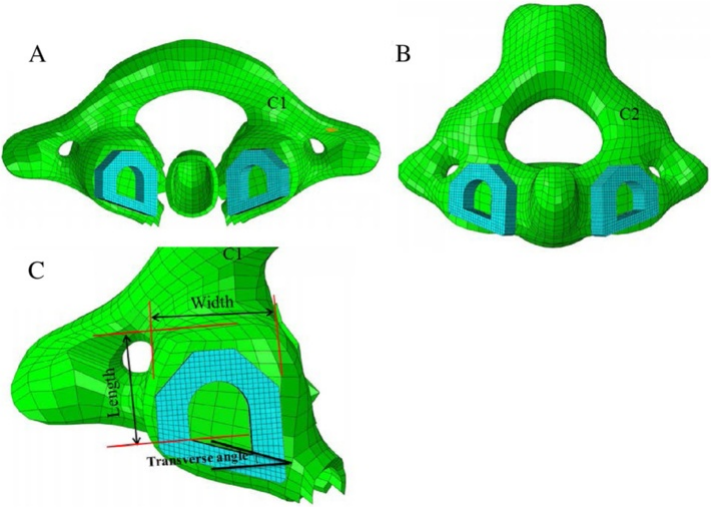
The novel transoral atlantoaxial fusion cage. **a** Inferior aspect of the cage on atlas; **b** Superior aspect of the cage on axis; **c** The hexagon-shaped cage with its width, length, and transeverse angle

Before designing the transoral atlantoaxial fusion cage, an anatomic study the atlantoaxial lateral mass was necessary. Several authors have addressed the quantitative anatomical and morphometric of the atlantoaxial lateral mass [28, 29]. The mean diameter of the inferior lateral mass of the atlas (16.8 ± 1.7 mm) is somewhat smaller than the diameter of the lateral mass of the axis (17.7 ± 1.5 mm), which results in the surface areas of the inferior lateral mass of atlas (211.8 ± 35.4 mm^2^) are slightly smaller than that of the superior lateral mass of axis (234.8 ± 39.8 mm^2^) [29]. Thus, the diameter of the self-designed cage is based on the diameter of the lateral mass of C1 rather than that of C2 (Fig. 3a-b). Dong et al. [28] measured the transverse diameter and longitudinal diameters of the lateral mass of C1 were 17.90 ± 1.18 mm and 15.63 ± 1.04 mm, respectively. Similarly, Cattrysse et al. [29] found those diameters to be 17.2 ± 2.0 mm and 16.6 ± 1.6 mm for C1. As for the height of cage, a morphometric computed tomography study by Li et al. [30] reported the mean interval of atlantoaxial lateral mass was 3.0 ± 0.5 mm (range 2.1–4.8 mm). Finally, another practical consideration is the slope of the anterior surface of the atlas, which is variable with the transverse angle in the axial plane from 16° to 28° [31]. Considering anatomical feature of the atlantoaxial lateral mass as mentioned above, the transoral fusion cage was designed with asymmetrical hexagon shape to accommodate the anatomy of the atlantoaxial joint; this design is meant to not only avoid injury of the spinal cord, VA and internal carotid artery, but also to provide large internal graft window between bone graft and endplate. The transoral fusion cage size used in this study was constructed to a length of 12 mm, width of 12 mm, height of 5 mm and transverse angle of 19° (Fig. 3c), taking into consideration the size of the C1-C2 joint used in this study; however, the various other sizes of cage are available to meet individual requirements.

The Cage + Plate device consists of the hexagon-shaped cage with an additional connected titanium plate and two diverging titanium screws that are fixed to the superior C1 endplate and inferior C2 interarticularis; this design is meant to provide strong support and fixation to an unstable spine so that an additional posterior fusion would not be required.

### The advantages of transoral atlantoaxial fusion cage

The transoral atlantoaxial fusion cage offers several advantages to address. 1. Clinical studies have demonstrated that the contraction of anterior muscles, ligaments, and capsules of atlantoaxial joint, especially the osteophytes and scar tissue inside the atlanto-dens interspace prevent the reduction in BI with IAAD [4, 6–9], thus most cases can achieve anatomic reduction duiring transoral release procedure. After removing the articular surface cartilage, the C1-2 joint is completely loosened and the lateral mass of C1 and C2 can be separated by approximately 5–10 mm [4], so that inserting a transoral cage is easy to pull the den downward to decompress the ventral cord directly. 2. Clinical studies have demonstrated that the C0–C2 angle had a significant negative correlation with the C2–C7 angle [32–34], and surgical correction of the C0-C2 deformity in patients with IAAD to within the range of physiological lordosis results in a secondary correction of C2-C7 alignment to within the range of physiological lordosis [32], therefore it is logical to think that these changes can also be found between C1–C2 angle and C2–C7 angle. Normal values for sagittal alignment in asymptomatic individuals have been established that the mean C1–C2 angle are 28.2 ± 4.0° in females significantly larger than 26.4 ± 4.6° in males, and C2–C7 angles are 12.7 ± 6.6° and 16.3 ± 7.3°, correspondingly [33]. It is crucial to restore the C0–C2 angle or C1–C2 angle within the normal range at the time of surgery because misalignment of the upper cervical spine can lead to postoperative dyspnea and/or dysphagia [35, 36]. Thus, the goal of treatment of BI with IAAD is not only to provide neural decompression, stabilization and fusion, but also to restore C1–C2 fusion angle. In case of BI with slope shape of articular surface of C2 [37], a wedge-shaped cage placement enable correction of such deformity and maintain atlantoaxial fusion angle. Therefore, cages with different shapes are available, to be adaptable to the different abnormalities. 3. The transoral cage, which exceeds the physiological height of C1-C2 joint, can improve stability to the C1-C2 complex because of the increased tension of ligamentous structures of the C1-C2 joint [30, 38]. 4. The transoral cage filled with bone graft, which has the inherent properties of osteoconduction and osteoinduction, provides a load bearing surface area for atlantoaxial fusion, and may prevent bone graft collapse, extrusion, resorption and micromotion.

### ROM data

In recent years, the 2-screw or 4-screw anchored cage device is widely used for anterior cervical discectomy and fusion (ACDF), and maintain the cervical lordosis and disk height with a very low incidence of postoperative dysphagia [11, 39–41]. A study by Kasliwal et al. [11] reported satisfactory clinical and radiographic outcomes with 2-screw anchored cage for ACDF. Only 13 % patients had very low incidence of early postoperative dysphagia with 0 % incidence of dysphgia at 3 months. Qi et al. [41] compared the incidence of dysphagia in patients undergoing ACDF using 4-screw anchored cage and traditional anterior plate plus cage, and found a decreased incidence of dysphagia post-operatively with anchored cage. Biomechanically, Majid et al. [42] demonstrated that using 2-screw anchored cage for one-level ACDF had biomechanical stability comparable to that of traditional anterior plate plus cage. Similarly, Clavenna et al. [43] evaluated the biomechanical stability of the 2-screw anchored cage and showed that it provided stabilization comparable to traditional cage plus plate for two-level and three-level ACDF. In contrast, another biomechanical study reported in 2014 by Reis et al. [44] showed that the 2-screw anchored cage provided similar biomechanical stability in lateral bending but lower stability in flexion, extension, and axial rotation compared with traditional anterior plate plus cage or a 4-screw anchored cage. The opposite findings may be attributed to the different plate and cage designs and bone quality of the specimens used in biomechanical studies.

**Fig. 4:**
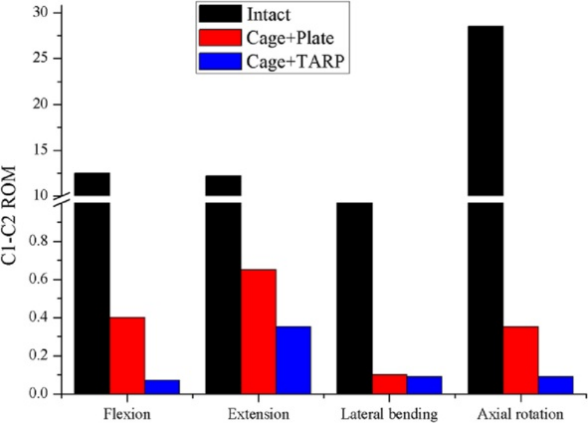
Comparison of C1-C2 ROM in the Cage + Plate and Cage + TARP models under vertical load of 40 N and torque of 1.5 Nm

The transoral anchored cage device is designed based on the 2-screw anchored cage, however, the biomechanics of the C1-2 joint are different from the subaxial cervical spine, the biomechanical properties of this novel device remain unclear. Our FE results indicate that the Cage + TARP model reduced the ROM by 82.5 %, 46.2 %, 10.0 % and 74.3 % in flexion, extension, lateral bending, and axial rotation, respectively, compared with Cage + Plate model (Fig. 4). This indicates that the Cage + Plate device may provide similar stability in lateral bending but lower stability in flexion, extension, and axial rotation in comparison to the Cage + TARP device. It is generally accepted that a higher degree of immobilization leads to higher fusion rates. Due to concern for decreased biomechanical stability of the Cage + Plate device, especially in flexion, extension and axial rotation, additional external immobilization after surgery would be recommended for patients with BI treated with this anchored cage.

Although the Cage + Plate device had the weaker ROM control of flexion, extension, and axial rotation, it may offer several advantages over the Cage + TARP device. Firstly, it may lower the occurrence and severity of dysphagia since its components are all contained within the C1-C2 joint, which prevents potential irritation upon oropharyngeal soft tissue. Secondly, it facilitates a less longitudinal incision at the median posterior pharyngeal wall to expose the C1–2 joint and there is no need to widely expose the anterior surface of the C1 and C2 vertebral body. Furthermore, its lag screw technique is to be applied to compress the space between superior and inferior surfaces of bone graft inside the cage and the relevant vertebral endplate for bone remodeling.

### Cage subsidence

**Fig. 5:**
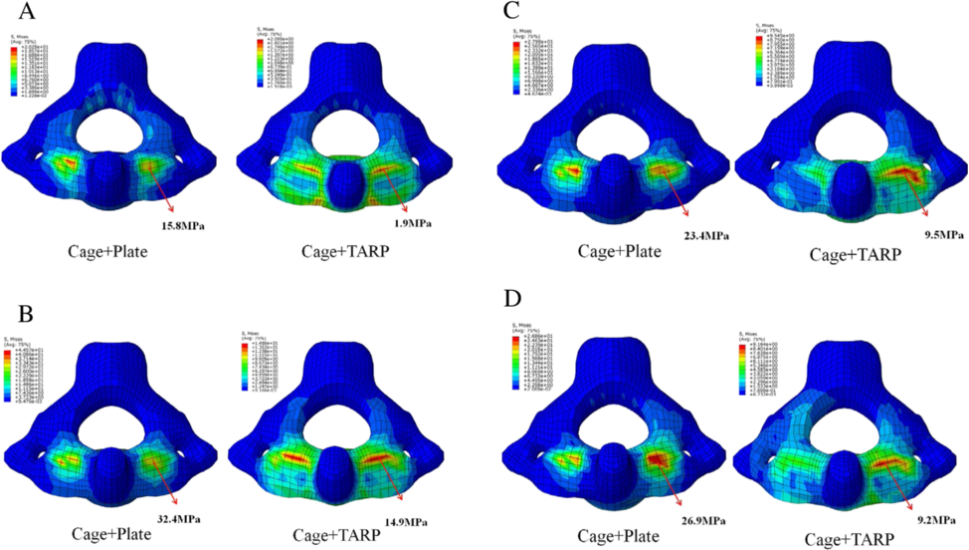
Maximum von Mises stress and stress distribution of the C2 endplate in the Cage + Plate and Cage + TARP models when tested with **a** flexion, **b** extension, **c** lateral bending and **d** axial rotation after applying a vertical load of 40 N

Subsidence is inherent in the interbody fusion processand is defined as sinking of a body with a higher elasticity modulus (eg, graft, cage, spacer) into a body characterized by a lower elasticity modulus (eg, vertebral body), resulting in changes in spinal geometry [45]. Some studies have evaluated the rate of cage subsidence following ACDF with the anchored cage device, at single or multiple levels. Njoku et al. [39] reported that 4-screw anchored cage subsidence of greater than 3 mm occurred in 15 of 66 operated levels (22.7 %) at a mean 9.8-month follow-up, with an average subsidence of 4.1 ± 4.7 mm. Kasliwal et al. [11] reported that 2-screw anchored cage subsidence occurred in 16 of 20 operated levels (80 %) at a mean 10-month follow-up, but none of them had a subsidence > 2 mm with the average subsidence of 1.1 mm (range: 0.4-2 mm). However, both Njoku [39] and Kasliwal [11] reported that none of them had subsidence-related symptoms that required revision surgery since postoperative cervical lordosis was radiologically maintained throughout follow-up. Similarly, Kao et al. [46] also concluded that subsidence was not associated with clinical and radiological outcomes but associated with more disc height change. Spacer or cage has been used to reduce the BI and achieve atlantoaxial fusion through posterior approach [1, 2, 10], however, the authors did not investigate spacer or cage subsidence. Yoshizumi et al. [10] treated a patient with BI using a cylindrical titanium cage packed with bone graft for atlantoaxial distraction and fusion and suggested that clinical observation about alignment change and cage subsidence would be continued over the long follow-up. The C2 endplate in the Cage + Plate model, which sustained 2.2 to 8.3 times greater stress than in Cage + TARP model, had higher stress than that in Cage + TARP model in all motions (Fig. 5). Increased stress on C2 endplate in Cage + Plate might result in high risk of cage subsidence, however, the MVMS of the C2 endplate in Cage + Plate model was 32.4 MPa occurred in extension, below the yield strength of the endplate of the cervical vertebrae (mean range of 104–208 MPa) [47]. There are no studies referred to the transoral cage for C1-C2 fusion, and further studies are required to assess the relationship beween transoral cage subsidence and clinical and radiological outcomes.

### Bone graft stresses

**Fig. 6:**
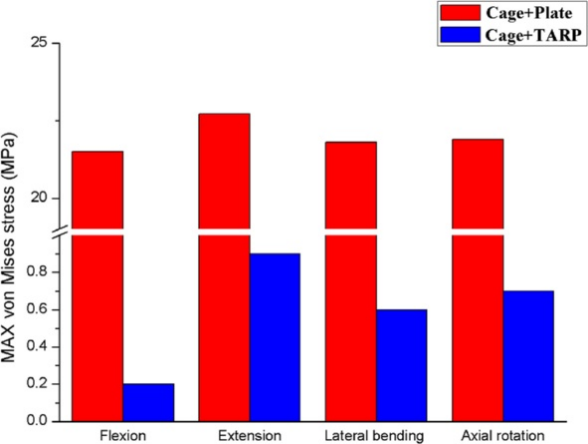
Bone graft stress in the Cage + Plate and Cage + TARP models

According to the Wolff law, load bearing plays a significant role in bone remodeling and maintenance of bone mass. Animal studies have showed that excessive stress shielding could inhibit fusion [48, 49]. Thus, transmission of stress to the bone graft within the cage was important for fusion and remodeling. The MVMS of the bone graft was found to be high in Cage + Plate model in all loading conditions (Fig. 6), particularly in flexion, the bone graft in Cage + Plate sustained 107.5 times greater stress than in Cage + TARP model. The MVMS of the bone graft in Cage + TARP model was only 1.9 MPa as the stress was shielded by the screws of the TARP device. Our FE analysis indicates that the Cage + Plate device may enhance load sharing ability and reduce the bone graft stress shielding and thus provides more favorable conditions for successful fusion.

### Study limitations

Our study had several potential limitations. First, with clinical BI, there is pathological vertical settling, presumably as the result of deformation of the bones and laxity of the ligaments [38]. However, the unstable BI model via removing all transverse ligament elements did not closely approximate this condition. It is challenging to model various conditions of BI. Second, the FE study used tie contact between screw and surrounding bone, and also made perfect surface-to-surface contact between cage and bony endplate. This assumption would result in a smaller ROM than that in clinical trial. Also, the cage is likely to have variations with respect to shape, length, width, height and its orientation. Changes in size and position of the cage may led to different results in terms of ROM and stress distribution. Finally, the FE study was also limited by the use of linear elastic and homogeneous materials for the entire vertebral body and ligaments, which are actually nonlinear characteristics in vitro and in vivo.

## Conclusions

Both the Cage + Plate and Cage + TARP devices not only provide neural decompression, stabilization and fusion, but also restore realignment of the upper cervical spine through a solely transoral approach without the need of a posterior operation. Our FE analysis results indicate that the novel Cage + Plate device may provide similar biomechanical stability in lateral bending but lower stability in flexion, extension, and axial rotation compared with Cage + TARP device and thus it may be utilized with additional external immobilization for patients with BI and IAAD. This anchored cage device may reduce stress shielding of the bone graft for successful fusion and minimize the risk of postoperative dysphagia. Clinical trials are now required to validate the reproducibility and advantages of our findings using this anchored cage for the treatment of BI with IAAD.

## Abbreviations

TARP: Transoral atlantoaxial reduction plate
BI: Basilar invagination
IAAD: Irreducible atlantoaxial dislocation
FEM: Finite element model
ROM: Range of motion
MVMS: Maximum von mises stress
